# Bundle Block Adjustment with 3D Natural Cubic Splines

**DOI:** 10.3390/s91209629

**Published:** 2009-12-02

**Authors:** Won Hee Lee, Kiyun Yu

**Affiliations:** Department of Civil and Environmental Engineering, Seoul National University, 599 Gwanak-ro, Gwanak-gu, Seoul 151-742, Korea; E-Mail: wlee2432@snu.ac.kr

**Keywords:** bundle block adjustment, 3D natural cubic splines, arc-length parameterization, linear features, line photogrammetry

## Abstract

Point-based methods undertaken by experienced human operators are very effective for traditional photogrammetric activities, but they are not appropriate in the autonomous environment of digital photogrammetry. To develop more reliable and accurate techniques, higher level objects with linear features accommodating elements other than points are alternatively adopted for aerial triangulation. Even though recent advanced algorithms provide accurate and reliable linear feature extraction, the use of such features that can consist of complex curve forms is more difficult than extracting a discrete set of points. Control points that are the initial input data, and break points that are end points of segmented curves, are readily obtained. Employment of high level features increases the feasibility of using geometric information and provides access to appropriate analytical solutions for advanced computer technology.

## 1. Introduction

One of the major tasks in digital photogrammetry is to determine the orientation parameters of aerial images quickly and accurately, which involves the two primary steps of interior and exterior orientation. While the original aerial photography provides the interior orientation parameters, the problem remains to determine the exterior orientation with respect to the object coordinate system. Exterior orientation establishes the position of the camera projection center in the ground coordinate system and the three rotation angles of the camera axis represent the transformation between the image and the object coordinate system. Exterior orientation parameters (EOPs) for a stereo model consisting of two aerial images can be obtained using relative and absolute orientation. This is a fundamental task in many applications such as surface reconstruction, orthophoto generation, image registration, and object recognition. The EOPs of multiple overlapping aerial images can be computed using a bundle block adjustment. The position and orientation of each exposure station are obtained by bundle block adjustments using collinearity equations that are linearized as having an unknown position and orientation with the object space coordinate system.

The program for bundle block adjustment in most softcopy workstations employs point features as the control information. Photogrammetric triangulation using digital photogrammetric workstations is more automated than aerial triangulation using analog instruments because the stereo model can be directly set using analytical triangulation outputs. Bundle block adjustment reduces the cost of field surveying in difficult areas and verifies the accuracy of field observations during the adjustment process. Even though each stereo model requires at least two horizontal and three vertical control points, this method can reduce the number of control points with accurate orientation parameters. EOPs of all the photographs in the target area are determined by the adjustment, which improves the accuracy and reliability of photogrammetric tasks. Because object reconstruction is processed by an intersection employing more than two images, bundle block adjustment provides the redundancy for the intersection geometry and contributes to the elimination of the gross error in the recovery of EOPs.

A stereo model consisting of two images with 12 EOPs is a common orientation unit. The mechanism of object reconstruction from a stereo model is comparable with that of an animal or human visual system. The principle aspects of the human vision system, including its neurophysiology, anatomy, and visual perception, are well described in Schenk [1]. A point-based procedure relationship between point primitives is widely developed in traditional photogrammetry, such that one measured point on an image is identified in another image. Even for linear features, data for a stereo model in a softcopy workstation is collected as points so that further application and analysis relies on points as primary input data units. The coefficients of interior, relative, and absolute orientation are computed from the point relationship. Interior orientation compensates for lens distortion, film shrinkage, scanner error, and atmosphere refraction. Relative orientation makes the stereoscopic view possible, and the relationship between a model coordinate system and an object space coordinate system is reconstructed by absolute orientation. Ground control points (GCPs) are widely employed to compute orientation parameters. Although the use of many GCPs is a time-consuming procedure and inhibits the robust and accurate automation that research into digital photogrammetry aims to achieve, the deployment of a computer, storage capacity, photogrammetric software, and a digital camera can reduce the computational and time complexity.

Employing high level features increases the feasibility of gaining geometric information and provides a suitable analytical situation for advanced computer technology. With advancing development in the extraction, segmentation, classification, and recognition of features, the input data for feature-based photogrammetry has been expanded at the expense of a redundancy in the application of aerial triangulation. Because the identification, formulation, and application of reasonable linear features is a crucial procedure for autonomous photogrammetry, higher order geometric feature-based modeling plays an important role in modern digital photogrammetry. The digital image format is suited to this purpose, especially in feature extraction and measurement, and it is useful for precise and rigorous modeling of features from images.

## 2. Line Photogrammetry

### 2.1. Overview of Line Photogrammetry

Line photogrammetry refers to applications such as single photo resection, relative orientation, triangulation, image matching, image registration, and surface reconstruction, which are implemented using linear features and the correspondence between linear features rather than points. Interest conjugate points such as edge points, corner points, and points on parking lanes operate well for determining EOPs with respect to the object space coordinate frame in traditional photogrammetry. The most well-known edge and interest point detectors are the Canny [2], Förstner [3], Harris, otherwise well-known as the Plessy detector [4], Moravec [5], Prewitt [6], Sobel [7], and SUSAN [8] detectors. The Canny, Prewitt, and Sobel operators are edge detectors and the Förstner, Harris, and SUSAN operators are corner detectors. Other well-known corner detection algorithms are the Laplacian of Gaussian, the difference of Gaussians, and the determinant of Hessian. Interest point operators that detect well-defined points, edges, and corners play an important role in automated triangulation and stereo matching. For example, the Harris operator is defined as a measurement of corner strength as:

(1) $$H(x,y)=\det(M)-\alpha {\left(\text{trace}(M)\right)}^{2}$$

where *M* is the local structure matrix and *α* is a parameter so that 0 ≤ *α* ≤ 0.25. A default value is 0.04. The gradients of the *x* and *y* directions are:

(2) $$g_{x}=\frac{\partial I}{\partial x},g_{y}=\frac{\partial I}{\partial y}$$

with *I* as an image. The local structure matrix *M* is:

(3) $$M=\left[\begin{array}{cc} A & C\\ C & B \end{array}\right]$$

where $A=g_{x}^{2}$, $B=g_{y}^{2}$ and *C* = *g_x_g_y_*.

A corner is detected when:

(4) $$H(x,y)>H_{\text{thr}}$$

where *H_thr_* is the threshold parameter on corner strength. The Harris operator searches points where variations in two orthogonal directions are large using the local autocorrelation function and provides good repeatability under varying rotation, scale, and illumination. The Förstner corner detector is also based on the covariance matrix for the gradient at a target point.

Marr [9] proposes the zero-crossing edge point detector utilizing second order rather than first order directional derivatives. The maximum of first order derivatives indicates the location of an edge whereas it is the zero of second order derivatives that indicates an edge. Physical boundaries of objects are easily detected because the gray levels change abruptly in boundaries. Because no single operator exists in edge detection, several criteria are required for each specific application. Matching point features present large percentages of match errors because point features are ambiguous and an analytical solution for point matching is not yet developed. Because of the geometric information and symbolic meaning of linear features, matching them is more reliable than matching point features in the autonomous environment of digital photogrammetry. As the use of linear features does not require a point-to-point correspondence, the matching of linear features is more flexible than that for points.

A number of researchers have published studies on automatic feature extraction and its application for various photogrammetric tasks: Förstner [10], Hannah [11], Schenk *et al.* [12], Schenk and Toth [13], Ebner and Ohlhof [14], Ackerman and Tsingas [15], Haala and Vosselman [16], Drewniok and Rohr [17,18], Zahran [19], and Schenk [20]. However, point-based photogrammetry based on manual measurement and the identification of interest points is not compatible with the autonomous environment of digital photogrammetry, but is a labor-intensive interpretation with the limitations of occlusion, ambiguity, and semantic information when compared with appropriate robust automation.

Because point features do not provide explicit geometric information, geometrical knowledge is achieved by perceptual organization [21-24]. Perceptual organization derives features and structures from imagery without a prior knowledge of the geometric, spectral, or radiometric properties of features and is a required step in object recognition. Perceptual organization is an intermediate level process for various vision tasks such as target-to-background discrimination, object recognition, target cueing, motion-based grouping, surface reconstruction, image interpretation, and change detection. Because objects cannot be distinguished by one gray level pixel, an image must be investigated entirely to obtain perceptual information. The most recent research related to perceptual organization concerns the 2D image implementation at signal, primitive, and structural levels.

In general, grouping or segmentation has the same meaning as perceptual organization in computer vision. This segmentation is typically addressed by two approaches, a model-based method (top-down approach) and a data-based method (bottom-up approach), and many researchers have employed edges and regions in segmentation. In the edge-based approaches, edges are likened to general forms of linear features without discontinuities, and in region-based approaches, iterative region growing techniques using seed points are preferred for surface fitting. Model-based methods require domain knowledge for each specific application in a manner similar to the human visual system, whereas data-based methods employ data properties for data recognition in a global fashion. In data-based methods, the same invariant properties in different positions and orientations are combined into the same regions or the same features. One approach alone, however, cannot guarantee consistent quality so combined approaches are implemented to minimize error segmentation.

Symbolic representation using distinct points is difficult because interest points contain no explicit information about physical reality. While traditional photogrammetric techniques obtain the camera parameters from the correspondence between 2D and 3D points, a more general and reliable process is required for advanced computer technology such as the adoption of linear features. Line photogrammetry is superior in higher level tasks such as object recognition and automation as compared with point-based photogrammetry, but selection of the correct candidate linear features is a complicated process. The development of the algorithm from point- to line-based photogrammetry uses the advantages of both approaches. The selection of suitable features is easier than the extraction of straight linear features and the candidate feature can be used in higher applications. A reason for developing curve features is that they will be prior to, and a fundamental aspect of, the next highest features such as surfaces, areas, and 3D volumes that consist of free-form linear features. Line-based photogrammetry is most suitable in the development of robust and accurate techniques for automation. If linear features are employed as control features, they provide advantages over points in the automation of aerial triangulation. Photogrammetry based on the manual measurement and identification of conjugate points is less reliable than line-based photogrammetry because it has the limitations of occlusion (visibility), ambiguity (repetitive patterns), and semantic information when considering the need for reliable and effective automation. The manual identification of corresponding entities within two images is crucial in the automation of point based photogrammetric tasks. No knowledge of the point-to-point correspondence is required in line-based photogrammetry. In addition, point features do not carry information about the scene whereas linear features contain the semantic information related to real object features. Additional information concerning linear features can increase the redundancy of the point system.

### 2.2. Literature Review

A review of related works begins with those using methods of pose estimation in imagery based on linear features that appear in most man-made objects such as buildings, roads, and parking lots. Over the years, a number of researchers in photogrammetry and computer vision have used line instead of point features; for example, Masry [25], Heikkila [26], Kubik [27], Petsa and Patias [28], Gülch [29], Wiman and Axelsson [30], Chen and Shibasaki [31], Habib [32], Heuvel [33], Tommaselli [34], Vosselman and Veldhuis [35], Förstner [36], Smith and Park [37], Schenk [38], Tangelder *et al.* [39], and Parian and Gruen [40]. Mulawa and Mikhail [41] originally proved the feasibility of linear features for close-range photogrammetric applications such as space intersection and resection, and relative and absolute orientation. This was the first step in employing linear feature-based methods in close-range photogrammetric applications. Mulawa [42] later developed linear feature-based methods for different sensors.

Whereas straight linear features and conic sections can be represented as unique mathematical expressions, free-form lines in nature cannot be described by algebraic equations. Hence, Mikhail and Weerawong [43] used splines and polylines to represent free-form lines as analytical expressions. Tommaselli and Tozzi [44] proposed that the accuracy of the straight line parameter be a subpixel with the representation of four degrees of freedom in an infinite line. Many researchers in photogrammetry have described straight lines as infinite lines using minimal representation to reduce unknown parameters. The main consideration in straight line expression is in the singularities. Habib *et al.* [45] made a bundle block adjustment using a 3D point set lying on control linear features instead of traditional control points. EOPs were reconstructed hierarchically employing automatic single photo resection (SPR).

Habib *et al.* [46] summarized linear features extracted from a mobile mapping system, a GIS database, and maps for various photogrammetric applications such as SPR, triangulation, digital camera calibration, image matching, 3D reconstruction, image to image registration, and surface to surface registration. In their work, matched linear feature primitives were utilized in space intersection for the reconstruction of object space features, and linear features in the object space were used as control features in triangulation and digital camera calibration.

Mikhail [47] and Habib *et al.* [48] accomplished the geometrical modeling and the perspective transformation of linear features within a triangulation process. Linear features were used to recover relative orientation parameters. Habib *et al.* proposed a free-form line in object space by a sequence of 3D points along the object space line.

Schenk [49] extended the concept of aerial triangulation from point features to linear features. The line equation of six dependent parameters replaced the point-based collinearity equation:

(5) $$\begin{array}{l} X=X_{A}+t\cdot a\\ Y=Y_{A}+t\cdot b\\ Z=Z_{A}+t\cdot c \end{array}$$

where a real variable is *t*, the start point (*X_A_,Y_A_,Z_A_*), and the direction vector (*a,b,c*).

The traditional point-based collinearity equation was extended to line features:

(6) $$\begin{array}{l} x_{p}=-f\frac{(X_{A}+t\cdot a-X_{C})r_{11}+(Y_{A}+t\cdot b-Y_{C})r_{12}+(Z_{A}+t\cdot c-Z_{C})r_{13}}{(X_{A}+t\cdot a-X_{C})r_{31}+(Y_{A}+t\cdot b-Y_{C})r_{32}+(Z_{A}+t\cdot c-Z_{C})r_{33}}\\ y_{p}=-f\frac{(X_{A}+t\cdot a-X_{C})r_{21}(Y_{A}+t\cdot b-Y_{C})r_{22}(Z_{A}+t\cdot c-Z_{C})r_{23}}{(X_{A}+t\cdot a-X_{C})r_{31}(Y_{A}+t\cdot b-Y_{C})r_{22}+(Z_{A}+t\cdot c-Z_{C})r_{33}} \end{array}$$

with *x_p_,y_p_* as photo coordinates, *f* the focal length, *X_C_,Y_C_,Z_C_* the camera perspective center, and *r_ij_* the elements of the 3D orthogonal rotation matrix. The extended collinearity equation with six parameters was derived as the line expression of four parameters (*ô,θ,x*_0_,*y*_0_) because a 3D straight line has only four independent parameters. Two constraints are required to solve a common form of the 3D straight equations using six parameters determined by two vectors:

(7) $$\left[\begin{array}{l} X\\ Y\\ Z \end{array}\right]=\left[\begin{array}{c} \cos\theta \cos\phi \cdot x_{0}-\sin\phi \cdot y_{0}+\sin\theta \cos\phi \cdot z\\ \cos\theta \sin\phi \cdot x_{0}+\cos\phi \cdot y_{0}+\sin\theta \sin\phi \cdot z\\ -\sin\theta \cdot x_{0}+\cos\theta \cdot z \end{array}\right]$$

where *z* is a real variable. The advantage of the 3D straight line using four independent parameters is that it reduces the computation and time complexity in processes such as bundle block adjustment. The collinearity equation as the straight line function of four parameters was developed:

(8) $$\begin{array}{l} x_{p}=-f\frac{(X-X_{C})r_{11}(Y-Y_{C})r_{12}+(Z-Z_{C})r_{13}}{(X-X_{C})r_{31}+(Y-Y_{C})r_{32}+(Z-Z_{C})r_{33}}\\ y_{p}=-f\frac{(X-X_{C})r_{21}+(Y-Y_{C})r_{22}+(Z-Z_{C})r_{23}}{(X-X_{C})r_{31}+(Y-Y_{C})r_{32}+(Z-Z_{C})r_{33}} \end{array}$$

where *X, Y*, and *Z* were defined in equation (7).

Zalmanson [50] updated EOPs using the correspondence between the parametric control free-form line in object space and the projected 2D free-form line in image space. The hierarchical approach, the modified iteratively close point (ICP) method, was developed to estimate curve parameters. The ray lies on the free-form line whose parametric equation represented by one parameter follows. Besl and McKay [51] employed the ICP algorithm to solve a matching problem of point sets, free-form curves, surfaces, and terrain models in 2D and 3D space. In their work, an ICP algorithm was executed without prior knowledge of the correspondence between points. The ICP method affected the Zalmanson's dissertation on the development of the recovery of EOPs using 3D free-form lines in photogrammetry. Euclidean 3D transformation was then employed in a search for the closest entity in the geometric data set. Rabbani *et al.* [52] utilized the ICP method in the registration of Lidar point clouds to divide them into four categories (spheres, planes, cylinders, and tori) by direct and indirect methods.

(9) $$\Xi (l)=\left[\begin{array}{c} X(l)\\ Y(l)\\ Z(l) \end{array}\right]=\left[\begin{array}{c} X_{0}^{k}\\ Y_{0}^{k}\\ Z_{0}^{k} \end{array}\right]+\left[\begin{array}{c} \rho_{1}\\ \rho_{2}\\ \rho_{3} \end{array}\right]l$$

where *X*_0_, *Y*_0_, *Z*_0_, *ω, φ, κ* are the EOPs and *ρ*_1_, *ρ*_2_, *ρ*_3_ are the direction vector.

The parametric curve Γ(*t*) = [*X*(*t*) *Y*(*t*) *Z*(*t*)]*^T^* was obtained by minimizing the Euclidian distance between two parametric curves:

(10) $$\Phi (t,l)\equiv {\left\|\Gamma (t)-\Xi (l)\right\|}^{2}={\left(X(t)-X_{0}-\rho_{1}l\right)}^{2}+{\left(Y(t)-Y_{0}-\rho_{2}l\right)}^{2}+{\left(Z(t)-Z_{0}-\rho_{3}l\right)}^{2}$$

Φ(*t,l*) had a minimum value at ∂Φ/∂*l* = ∂Φ/∂*t* = 0 with two independent variables *l* and *t* as in (11).

(11) $$\begin{array}{l} \partial \Phi /\partial l=-2\rho_{1}(X(t)-X_{0}-\rho_{1}l)-2\rho_{2}(Y(t)-Y_{0}-\rho_{2}l)-2\rho_{3}(Z(t)-Z_{0}-\rho_{3}l)=0\\ \partial \Phi /\partial t=2X^{\prime }(t)(X(t)-X_{0}-\rho_{1}l)+2Y^{\prime }(t)(Y(t)-Y_{0}-\rho_{2}l)+2Z^{\prime }(t)(Z(t)-Z_{0}-\rho_{3}l)=0 \end{array}$$

Akav *et al.* [53] employed planar free-form curves for aerial triangulation with the ICP method. Because the effect of the *Z* parameter as compared with that of *X* or *Y* was large in a normal plane equation *aX* + *bY* + *cZ* = 1, a different plane representation was developed to avoid numerical problems:

(12) $$\begin{array}{l} \left[\begin{array}{c} n_{1}\\ n_{2}\\ n_{3} \end{array}\right]=\left[\begin{array}{c} \sin\theta \cos\varphi \\ \sin\theta \sin\varphi \\ \cos\varphi \end{array}\right]\\ n_{1}(X-X_{0})+n_{2}(Y-Y_{0})+n_{3}(Z-Z_{0})=0\\ n_{1}X+n_{2}Y+n_{3}Z=D \end{array}$$

with *θ* the angle from the *XY* plane, *φ* the angle around the *Z* axis, *n* the unit vector of plane normal, and *D* the distance between the plane and the origin. Five relative orientation parameters and three planar parameters were obtained by using the homography mapping system, which searched for the conjugate point in an image corresponding to a point in the other image.

Lin [54] proposed the method of autonomous recovery of exterior orientation parameters by an extension of the traditional point-based modified iterated Hough transform (MIHT) to the 3D free-form linear feature-based MIHT. Straight polylines were generalized for matching primitives in the pose estimation because the mathematical representation of straight lines is much clearer than the algebraic expression of conic sections and splines.

Gruen and Akca [55] matched 3D curves whose forms were defined by a cubic spline using matching least squares. Subpixels were localized by the matching, and the quality of the localization was decided by the geometry of image patches. Two free-form lines were defined as:

(13) $$\begin{array}{l} f(u)={\left[\begin{array}{ccc} x(u) & y(u) & z(u) \end{array}\right]}^{T}=a_{0}+a_{1}u+a_{2}u^{2}+a_{3}u^{3}\\ g(u)={\left[\begin{array}{ccc} x^{\prime }(u) & y^{\prime }(u) & z^{\prime }(u) \end{array}\right]}^{T}=b_{0}+b_{1}u+b_{2}u^{2}+b_{3}u^{3} \end{array}$$

where *u* ∈ [0,1],*a*_0_, *a*_1_, *a*_2_, *a*_3_, *b*_0_, *b*_1_, *b*_2_, *b*_3_ are variables and the *f*(*u*), *g*(*u*) ∈ R^3^.

The Taylor expansion was employed to adopt the Gauss–Markov model:

(14) $$\begin{array}{l} f(u)-e(u)=g(u)\\ f(u)-e(u)=g^{0}(u)+\frac{\partial g^{0}(u)}{\partial u}du\\ f(u)-e(u)=g^{0}(u)+\frac{\partial g^{0}(u)}{\partial u}\frac{\partial u}{\partial x}dx+\frac{\partial g^{0}(u)}{\partial u}\frac{\partial u}{\partial y}dy+\frac{\partial g^{0}(u)}{\partial u}\frac{\partial u}{\partial z} \end{array}$$

#### 3. Bundle Block Adjustment with 3D Natural Cubic Splines

##### 3.1. 3D Natural Cubic Splines

The choice of the right feature model is important in the development of a feature-based approach because an ambiguous feature representation leads to unstable adjustment. A spline is a piecewise polynomial function in the *n* of vector graphics. Splines are widely used for data fitting in computer science because of the resultant simplicity in curve reconstruction. Complex figures are well approximated through curve fitting and a spline lends strength to the accuracy evaluation, data interpolation, and curve smoothing. One of the important properties of a spline is that it can easily be morphed. A spline represents a 2D or 3D continuous line within a sequence of pixels and segmentation. The relationship between pixels and lines is applied to a bundle block adjustment or a functional representation. A spline of degree 0 is the simplest spline, a linear spline has degree 1, a quadratic spline has degree 2, and a common natural cubic spline has degree 3 with continuity C2. The geometrical meaning of continuity *C^2^* is that the first and second derivatives are proportional at joint points and the parametric importance of continuity *C^2^* is that the first and second derivatives are equal at connected points.

The number of break points that are the determination of a set of piecewise cubic functions varies depending upon the spline parameters. A natural cubic degree guarantees a second-order continuity, which means that the first and second order derivatives of two consecutive natural cubic splines are continuous at the break point. The intervals for a natural cubic spline do not need to be the same as the distance of every two consecutive data points. The best intervals are chosen by a least squares method. In general, the total number of break points is less than that of original input points. The algorithm of a natural cubic spline is as below.

Generate the break point (control point) set for the spline of the original input data.
Calculate the maximum distance between the approximated spline and the original input data while the maximum distance >the threshold of the maximum distance.
Add the break point to the break point set at the location of the maximum distance.
Compare the maximum distance with the threshold.

A larger threshold makes for more break points with a more accurate spline to the original input data. Npiecewise cubic polynomial functions between two adjacent break points are defined from the N +1 break points. There is a separate cubic polynomial for each segment with its own coefficients.

(15) $$\begin{array}{l} X_{0}(t)=a_{00}+a_{01}t+a_{02}t^{2}+a_{03}t^{3},t\in \left[0,1\right]\\ X_{1}(t)=a_{10}+a_{11}t+a_{12}t^{2}+a_{13}t^{3},t\in \left[0,1\right]\\ \ldots \\ X_{i}(t)=a_{i0}+a_{i1}t+a_{i2}t^{2}+a_{i3}t^{3},t\in \left[0,1\right]\\ Y_{i}(t)=b_{i0}+b_{i1}t+b_{i2}t^{2}+b_{i3}t^{3},t\in \left[0,1\right]\\ Z_{i}(t)=c_{i0}+c_{i1}t+c_{i2}t^{2}+c_{i3}t^{3},t\in \left[0,1\right] \end{array}$$

The strength of this approach is that segmented lines represent a free-form line with analytical parameters. The number of break points is reduced and the input error should be absorbed by a mathematical model, especially in the expression of points on a straight line. A natural cubic spline is a data-independent curve fitting. The disadvantage is that the whole curve shape depends on all of the passing points, and changing any one of these alters the entire curve.

**Figure 1.**
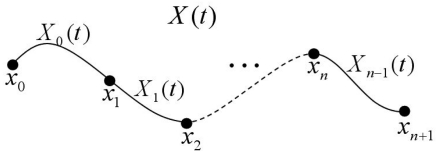
Natural cubic splines.

The correspondence between the 3D curve in the object space coordinate system and its projected 2D curve in the image coordinate system is implemented using an accommodating natural cubic spline curve feature because of its boundary conditions that retain zero second derivatives at the end points. A natural cubic spline is composed of a sequence of cubic polynomial segments as in Figure 1 with *x*_0_,*x*_1_,…,*x*_n_ as the *n* + 1 control points and *X*_0_,*X*_1_,…,*X*_n-1_ as the ground coordinates of *n* segments.

##### 3.2. Extended Collinearity Equation Model for Splines

Collinearity equations are the commonly used condition equations to determine relative orientation. The space intersection calculates a point location in object space using the projection ray intersection from two or more images, and the space resection determines the coordinates of a point on an image and the EOPs with respect to the object space coordinate system. The space intersection and the space resection are the fundamental operations in photogrammetry for further processes such as triangulation. The basic concept of the collinearity equation is that all points on the image, the perspective center, and the corresponding point in the object space are all on a straight line. The relationship between the image and object coordinate systems is expressed by three position parameters and three orientation parameters. Collinearity equations play an important role in photogrammetry because each control point in object space produces two collinearity equations for every photograph in which the point appears. If *m* points appear in *n* images, then *2mn* collinearity equations can be employed in the bundle block adjustment. The extended collinearity equations relating a natural cubic spline in object space with ground coordinates (*X_i_*(*t*),*Y_i_*(*t*),*Z_i_*(*t*)) with image space having photo coordinates (*x_pi_,y_pi_*) are seen as (16). A natural cubic spline allows the utilization of the collinearity model for expressing orientation parameters and curve parameters as below:

(16) $$\begin{array}{c} x_{pi}=-f\frac{(X_{i}(t)-X_{C})r_{11}+(Y_{i}(t)-Y_{C})r_{12}+(Z_{i}(t)-Z_{C})r_{13}}{(X_{i}(t)-X_{C})r_{31}+(Y_{i}(t)-Y_{C})r_{32}+(Z_{i}(t)-Z_{C})r_{33}}\\ y_{pi}=-f\frac{(X_{i}(t)-X_{C})r_{21}+(Y_{i}(t)-Y_{C})r_{22}+(Z_{i}(t)-Z_{C})r_{23}}{(X_{i}(t)-X_{C})r_{31}+(Y_{i}(t)-Y_{C})r_{32}+(Z_{i}(t)-Z_{C})r_{33}} \end{array}$$

with (*x_pi_,y_pi_*) as the photo coordinate, *f* the focal length, *X_C_,Y_C_,Z_C_* the camera perspective center, and *r_ij_* the elements of the 3D orthogonal rotation matrix *R^T^* by the angular elements (*ω,φ,κ*) of EOPs.

The extended collinearity equations can be written as follows:

(17) $$\begin{array}{l} x_{p}=-f\frac{u}{w},y_{p}=-f\frac{v}{w}\\ \left[\begin{array}{c} u\\ v\\ w \end{array}\right]=R^{T}(\omega ,\varphi ,\kappa )\left[\begin{array}{c} X_{i}(t)-X_{C}\\ Y_{i}(t)-Y_{C}\\ Z_{i}(t)-Z_{C} \end{array}\right] \end{array}$$

To recover the 3D natural cubic spline parameters and the exterior orientation parameters in a bundle block adjustment, a nonlinear mathematical model of the extended collinearity equation is differentiated. The models of exterior orientation recovery are classified into linear and nonlinear methods. Whereas linear methods decrease the computation load, the accuracy and reliability of linear algorithms are not excellent. Nonlinear methods are more accurate and predictable. However, nonlinear methods require initial estimates and they increase the computational complexity. The relationship between a point in image space and a corresponding point in object space is established by the extended collinearity equation. Prior knowledge of the correspondences between individual points in the 3D object space and their projected features in the 2D image space is not required in extended collinearity equations with 3D natural splines. One point on a cubic spline has 19 parameters (*X_C_,Y_C_,Z_C_,ω,φ,κ,a*_0_,*a*_1_,*a*_2_,*a*_3_,*b*_0_,*b*_1_,*b*_2_,*b*_3_,*c*_0_,*c*_1_,*c*_2_,*c*_3_,*t*). The differentials of (17) are derived by (18):

(18) $$dx_{p}=-\frac{f}{w}du+\frac{fu}{w^{2}}dw,dy_{p}=-\frac{f}{w}dv+\frac{fv}{w^{2}}dw$$

with differentials of du, *dv*, and *dw* (19).

(19) $$\begin{array}{ll} \left[\begin{array}{c} du\\ dv\\ dw \end{array}\right]= & \frac{\partial R^{T}}{\partial \omega }\left[\begin{array}{c} X_{i}(t)-X_{C}\\ Y_{i}(t)-Y_{C}\\ Z_{i}(t)-Z_{C} \end{array}\right]d\omega +\frac{\partial R^{T}}{\partial \varphi }\left[\begin{array}{c} X_{i}(t)-X_{C}\\ Y_{i}(t)-Y_{C}\\ Z_{i}(t)-Z_{C} \end{array}\right]d\varphi +\frac{\partial R^{T}}{\partial \kappa }\left[\begin{array}{c} X_{i}(t)-X_{C}\\ Y_{i}(t)-Y_{C}\\ Z_{i}(t)-Z_{C} \end{array}\right]d\kappa -R^{T}\left[\begin{array}{c} 1\\ 0\\ 0 \end{array}\right]dX_{C}\\ \; & -R^{T}\left[\begin{array}{c} 0\\ 1\\ 0 \end{array}\right]dY_{C}-R^{T}\left[\begin{array}{c} 0\\ 0\\ 1 \end{array}\right]dZ_{C}+R^{T}\left[\begin{array}{c} 1\\ 0\\ 0 \end{array}\right]da_{0}+R^{T}\left[\begin{array}{c} t\\ 0\\ 0 \end{array}\right]da_{1}+R^{T}\left[\begin{array}{c} t^{2}\\ 0\\ 0 \end{array}\right]da_{2}+R^{T}\left[\begin{array}{c} t^{3}\\ 0\\ 0 \end{array}\right]da_{3}+R^{T}\left[\begin{array}{c} 0\\ 1\\ 0 \end{array}\right]db_{0}\\ \; & -R^{T}\left[\begin{array}{c} 0\\ t\\ 0 \end{array}\right]db_{1}-R^{T}\left[\begin{array}{c} 0\\ t^{2}\\ 0 \end{array}\right]db_{2}+R^{T}\left[\begin{array}{c} 0\\ t^{3}\\ 0 \end{array}\right]db_{3}+R^{T}\left[\begin{array}{c} 0\\ 0\\ 1 \end{array}\right]dc_{0}+R^{T}\left[\begin{array}{c} 0\\ 0\\ t \end{array}\right]dc_{1}+R^{T}\left[\begin{array}{c} 0\\ 0\\ t^{2} \end{array}\right]dc_{2}+R^{T}\left[\begin{array}{c} 0\\ 0\\ t^{3} \end{array}\right]dc_{3}\\ \; & +R^{T}\left[\begin{array}{c} a_{1}+2a_{2}t+3a_{3}t^{2}\\ b_{1}+2b_{2}t+3b_{3}t^{2}\\ c_{1}+2c_{2}t+3c_{3}t^{2} \end{array}\right]dt \end{array}$$

Substituting *du, dv*, and *dw* in (18) by the expressions found in (19) leads to:

(20) $$\begin{array}{l} dx_{p}=M_{1}dX_{C}+M_{2}dY_{C}+M_{3}dZ_{C}+M_{4}d\omega +M_{5}d\varphi +M_{6}d\kappa +M_{7}da_{0}+M_{8}da_{1}+M_{9}da_{2}+M_{10}da_{3}+M_{11}db_{0}+M_{12}db_{1}+M_{13}db_{2}+M_{14}db_{3}+M_{15}dc_{0}+M_{16}dc_{1}+M_{17}dc_{2}+M_{18}dc_{3}+M_{19}dt\\ dy_{p}=N_{1}dX_{C}+N_{2}dY_{C}+N_{3}dZ_{C}+N_{4}d\omega +N_{5}d\varphi +N_{6}d\kappa +N_{7}da_{0}+N_{8}da_{1}+N_{9}da_{2}+N_{10}da_{3}+N_{11}db_{0}+N_{12}db_{1}+N_{13}db_{2}+N_{14}db_{3}+N_{15}dc_{0}+N_{16}dc_{1}+N_{17}dc_{2}+N_{18}dc_{3}+N_{19}dt \end{array}$$

*M*_1_,…*M*_19_,*N*_1_,…*N*_19_ denotes the partial derivatives of the extended collinearity equation for curves. The linearized extended collinearity equations by Taylor expansion, ignoring the 2nd and higher order terms, can be written as follows:

(21) $$\begin{array}{l} x_{p}+f\frac{u^{0}}{w^{0}}=M_{1}dX_{C}+M_{2}dY_{C}+M_{3}dZ_{C}+M_{4}d\omega +M_{5}d\varphi +M_{6}d\kappa +M_{7}da_{0}+M_{8}da_{1}+M_{9}da_{2}+M_{10}da_{3}+M_{11}db_{0}+M_{12}db_{1}+M_{13}db_{2}+M_{14}db_{3}+M_{15}dc_{0}+M_{15}dc_{0}+M_{16}dc_{1}+M_{17}dc_{2}+M_{18}dc_{3}+M_{19}dt+e_{x}\\ y_{p}+f\frac{v^{0}}{w^{0}}=N_{1}dX_{C}+N_{2}dY_{C}+N_{3}dZ_{C}+N_{4}d\omega +N_{5}d\varphi +N_{6}d\kappa +N_{7}da_{0}+N_{8}da_{1}+N_{9}da_{2}+N_{10}da_{3}+N_{11}db_{0}+N_{12}db_{1}+N_{13}db_{2}+N_{14}db_{3}+N_{15}dc_{0}+N_{16}dc_{1}+N_{17}dc_{2}+N_{18}dc_{3}+N_{19}dt+e_{y} \end{array}$$

with *u*^0^,*v*^0^,*w*^0^ being the approximate parameters by $\left(X_{C}^{0},Y_{C}^{0},Z_{C}^{0},\omega^{0},\varphi^{0},\kappa^{0},a_{0}^{0},a_{1}^{0},a_{2}^{0},a_{3}^{0},b_{0}^{0},b_{1}^{0},b_{2}^{0},b_{3}^{0},c_{0}^{0},c_{1}^{0},c_{2}^{0},c_{3}^{0},t^{0}\right)$, and *e_x_,e_y_* the stochastic errors of *x_p_,y_p_*, the observed photo coordinates with zero expectation, respectively. Orientation parameters including the 3D natural cubic spline parameters are expected to recover correctly because the extended collinearity equations with these splines increase redundancy.

##### 3.3. Arc-Length Parameterization of 3D Natural Cubic Splines

The assumption made in bundle block adjustment by the Gauss–Markov model is that all the estimated parameters are uncorrelated. Hence, the design matrix of the adjustment must be full rank, nonsingular, and normal. However, because the spline parameters are not independent of their location parameters, additional observations are required to obtain parameter estimations. In a point-based approach, the point location relationship between image and object space is established for the pose estimation to include the fundamental camera position and orientation, the remote sensing, and the computer vision. The coordinates of a point are necessary for the space intersection and resection. To remove any rank deficiency caused by datum defects in point-based photogrammetry, some constraints are adopted to estimate the unknown parameters. The most common constraints are coplanarity, symmetry, perpendicularity, and parallelism. The minimum number of constraints is equal to the rank deficiency of the system. Inner constraints are often used in a photogrammetric network, which can be applied to both the object features and the camera orientation parameters. Angle or distance condition equations provide information on the relativity between observations in object space and points in image space. Absolute information can be obtained from the fixed control points.

In this research, an arc-length parameterization is applied as an additional condition equation to solve the rank deficient problem in extended collinearity equations using 3D natural cubic splines. The concept of differentiable parameterization is that the arc length of a curve can be divided into minute pieces and these can be summed such that each piece will be approximately linear. The sum of the squares of derivatives is the same with a velocity vector because a parametric curve can be considered as a point trajectory. A velocity vector describes the path of a curve and movement characteristics. If a particle on a curve moves at a constant rate, the curve is parameterized by the arc length. While the extended collinearity equation provides the only information, curves have additional geometric constraints such as arc length, tangent of location, and curvature. These support space resection under the assumption of properly accounting for additional independent observations in both the image and object space. Arc length in object space is determined by a geometric integration using a construction from the differentiable parameterization of a spline. Arc length in image space is calculated by a geometric integration of a construction from the differentiable parameterization of the photo coordinates derived from a spline in the object space:

(22) $$\begin{array}{ll} \text{Arc}(t) & =\int_{t_{i}}^{t_{i+1}}\sqrt{{\left(x_{p}^{\prime }(t)\right)}^{2}+{\left(y_{p}^{\prime }(t)\right)}^{2}dt}\\ \; & =\int_{t_{i}}^{t_{i+1}}\sqrt{{\left\{{\left(-f\frac{u(t)}{w(t)}\right)}^{\prime }\right\}}^{2}+{\left\{{\left(-f\frac{v(t)}{w(t)}\right)}^{\prime }\right\}}^{2}dt}\\ \; & =\int_{t_{i}}^{t_{i+1}}\sqrt{\left(-f\frac{u\prime (t)w(t)-u(t)w\prime (t)}{w^{2}(t)}\right)+{\left(-f\frac{v\prime (t)w(t)-v(t)w\prime (t)}{w^{2}(t)}\right)}^{2}dt} \end{array}$$

where *f* is the focal length and:

(23) $$\begin{array}{ll} \left[\begin{array}{c} u(t)\\ v(t)\\ w(t) \end{array}\right]=R^{T}(\omega ,\varphi ,\kappa ) & \left[\begin{array}{c} a_{0}+a_{1}t+a_{2}t^{2}+a_{3}t^{3}-X_{C}\\ b_{0}+b_{1}t+b_{2}t^{2}+b_{3}t^{3}-Y_{C}\\ c_{0+}c_{1}t+c_{2}t^{2}+c_{3}t^{3}-Z_{C} \end{array}\right]\\ \left[\begin{array}{c} u\prime (t)\\ v\prime (t)\\ w\prime (t) \end{array}\right]=R^{T}(\omega ,\varphi ,\kappa ) & \left[\begin{array}{c} a_{1}+2a_{2}t+3a_{3}t^{2}\\ b_{1}+2b_{2}t+3b_{3}t^{2}\\ c_{1}+2c_{2}t+3c_{3}t^{2} \end{array}\right] \end{array}$$

Because the problem of the arc-length parameterization of splines has no analytical solution, several numerical approximations of reparameterization techniques for splines or other curve representations have been developed. While most curves are not parameterized for arc length, the arc length of a B-spline can be reparameterized by adjusting its knots. Wang *et al.* [56] approximated the parameterized arc length of spline curves by generating a new curve that accurately approximated the original spline curve to reduce the computation complexity of the arc-length parameterization. They showed that the approximation of the arc-length parameterization works well in a variety of real-time applications including driving simulations.

Guenter and Parent [57] employed a hierarchical approach algorithm to develop a linear search arc-length subdivision table for parameterized curves to reduce the arc-length computation time. A table of the correspondence between parameter *t* and the arc length can be established to accelerate the arc-length computation. After dividing the parameter range into intervals, the arc length of each interval is computed for mapping parameters to the arc length. A reference table for various intervals of arc length can be developed. Another method of arc-length approximation is to use explicit functions such as the Bézier curve, which has advantages in fast function evaluations. Adaptive Gaussian integrations employ a recursive method that starts from a few samples and adds on more as necessary. Adaptive Gaussian integration also uses a table that maps curves or spline parameter values according to the arc-length values.

Nasri *et al.* [58] proposed an arc-length approximation method of circles and piecewise circular splines generated by control polygons or points using a recursive subdivision algorithm. While B-splines have various tangents over the curve depending upon the arc-length parameterization, circular splines have constant tangents whose vectors are useful in arc-length computation.

Simpson's rule is the numerical approximation of definite integrals. The geometric integration of the arc length in the image space can be calculated by this rule as follows:

(24) $$\begin{array}{ll} \text{Arc}(t) & =\int_{t_{1}}^{t_{2}}f(t)dt\\ \int_{t_{1}}^{t_{2}}f(t)dt & ≅\frac{t_{2}-t_{1}}{6}\left[f(t_{1})+4f\left(\frac{t_{2}+t_{1}}{2}\right)+f(t_{2})\right]\\ f(t) & =\sqrt{{\left(x_{p}^{\prime }(t)\right)}^{2}+{\left(y_{p}^{\prime }(t)\right)}^{2}}\\ \; & =\sqrt{{\left\{{\left(-f\frac{u(t)}{w(t)}\right)}^{\prime }\right\}}^{2}+{\left\{{\left(-f\frac{v(t)}{w(t)}\right)}^{\prime }\right\}}^{2}}\\ \; & =\sqrt{{\left(-f\frac{u\prime (t)w(t)-u(t)w\prime (t)}{w^{2}(t)}\right)}^{2}+{\left(-f\frac{v\prime (t)w(t)-v(t)w\prime (t)}{w^{2}(t)}\right)}^{2}}\\ & df=\frac{1}{2}{\left(f(t)\right)}^{\frac{1}{2}}f\left[2x_{p}^{\prime }(t)w\prime \frac{w^{2}}{w^{2}}du-2x_{p}^{\prime }(t)\frac{1}{w}du\prime +2y_{p}^{\prime }(t)\frac{w\prime }{w^{2}}dv-2y_{p}^{\prime }(t)\frac{1}{w}dv\prime +\left\{2x_{p}^{\prime }(t)\frac{u\prime w^{2}-(u\prime w-uw\prime )2w}{w^{4}}-2y_{p}^{\prime }(t)\frac{v\prime w^{2}-(v\prime w^{2}-vw\prime )2w}{w^{4}}\right\}dw+\left\{2x_{p}^{\prime }(t)\frac{u}{w^{2}}+2y_{p}^{\prime }\frac{v}{w^{2}}\right\}\text{Arc}(t)-\frac{t_{0}-t_{1}^{0}}{6}[f(t_{1}^{0})+4f\left(\frac{t_{2}^{0}+t_{1}^{0}}{2}\right)+f(t_{2}^{0})\right]\\ \; & =A_{1}dX_{C}+A_{2}dY_{C}+A_{3}dZ_{C}+A_{4}d\omega +A_{5}d\varphi +A_{6}d\kappa +A_{7}da_{0}+A_{8}da_{1}+A_{9}da_{2}+A_{10}da_{3}+A_{11}db_{0}+A_{12}db_{1}+A_{13}db_{2}+A_{14}db_{3}+A_{15}dc_{0}+A_{16}dc_{1}+A_{17}dc_{2}+A_{18}dc_{3}+A_{19}dt_{1}+A_{20}dt_{2}+e_{a} \end{array}$$

with $t_{1}^{0},t_{2}^{0},f(t^{0})$ being the approximate parameter using the following: $\left(X_{C}^{0},Y_{C}^{0},Z_{C}^{0},\omega^{0},\varphi^{0},\kappa^{0},a_{0}^{0},a_{1}^{0},a_{2}^{0},a_{3}^{0},b_{0}^{0},b_{1}^{0},b_{2}^{0},b_{3}^{0},c_{0}^{0},c_{1}^{0},c_{2}^{0},c_{3}^{0},t_{i}^{0}\right)$ and with *e_a_* being the stochastic error of the arc length between two locations with zero expectation. *A*_1_,…*A*_20_ denote the partial derivatives of the arc-length parameterization of a 3D natural cubic spline.

##### 3.4. Model Integration

The objective of bundle block adjustment is twofold, namely to calculate the exterior orientation parameters of a block of images and also the coordinates of the ground features in object space. In the determination of orientation parameters, additional interior conditions such as lens distortion, atmospheric refraction, and principal point offset can be obtained by self-calibration. In general, orientation parameters are determined by bundle block adjustment using a large number of control points. This establishment of control points, however, means expensive fieldwork, so an economical and accurate adjustment method is required. Linear features have several advantages to complement points in that they are useful for higher level tasks and they are easily extracted in man-made environments. The line photogrammetric bundle adjustment in this research aims at the estimation of exterior orientation parameters and 3D natural cubic spline parameters using the correspondence between splines in object space and spline observations of multiple images in image space. Nonlinear functions of orientation parameters, spline parameters, and spline location parameters are represented by extended collinearity and arc-length parameterization equations. Five observation equations are produced by each two points, and these are four extended collinearity equations (21) and one arc-length parameterization equation (24). An integrated model provides not only for the recovery of the image orientation parameters but also enables surface reconstruction using 3D curves. Of course, as the equation system of the integrated model has seven datum defects, control information about the coordinate system is required to obtain parameters. This is a step toward higher level vision tasks such as object recognition and surface reconstruction. In the case of straight lines and conic sections, tangents are additional observations in the integrated model. Conic sections, like points, provide good mathematical constraints because such sections provide nonsingular second degree equations. Such equations provide information for reconstruction and transformation and conic sections are divided by the eccentricity *e*. Because such sections can adopt more constraints than points and straight line features, they are useful for close range photogrammetric applications. In addition, conic sections have strength in correspondence establishment between 3D sections in object space and their counterpart features in 2D projected image space.

Ji *et al.* [59] employed conic sections for the recovery of EOPs, and Heikkila used them for camera calibration. A Hough transformation reduces the time complexity of conic section extraction using five parameter spaces for a SPR, camera calibration, and triangulation.

Parameters are linearized in the previous sections and the Gauss–Markov model is employed for the unknown parameter estimation. The equation system of the integrated model is described as:

(25) $$\begin{array}{c} \left[\begin{array}{lll} A_{\text{EOP}}^{k} & A_{SP}^{i} & A_{t}^{i}\\ \; & A_{AL}^{i} & \; \end{array}\right]\left[\begin{array}{c} \xi_{\text{EOP}}^{k}\\ \xi_{SP}^{i}\\ \xi_{i}^{i} \end{array}\right]=\left[y^{ki}\right]\\ A_{\text{EOP}}^{k}=\left[\begin{array}{cccc} M_{i1}^{k1} & M_{i2}^{k1} & \cdots & M_{i6}^{k1}\\ \; & \vdots & \; & \;\\ M_{i1}^{km} & M_{i2}^{km} & \cdots & M_{i6}^{km}\\ N_{i1}^{k1} & N_{i2}^{k1} & \cdots & N_{i6}^{k1}\\ \; & \vdots & \; & \;\\ N_{i1}^{km} & N_{i2}^{km} & \cdots & N_{i6}^{km} \end{array}\right]A_{SP}^{i}=\left[\begin{array}{cccc} M_{i7}^{1} & M_{i8}^{1} & \cdots & M_{i18}^{1}\\ \; & \vdots & \; & \;\\ M_{i7}^{m} & M_{i8}^{m} & \cdots & M_{i18}^{m}\\ N_{i7}^{1} & N_{i8}^{1} & \cdots & N_{i18}^{1}\\ \; & \vdots & \; & \;\\ N_{i7}^{m} & N_{i8}^{m} & \cdots & N_{i18}^{m} \end{array}\right]A_{t}^{i}=\left[\begin{array}{cccc} M_{i19}^{1} & M_{i20}^{1} & \cdots & M_{i18+n}^{1}\\ \; & \vdots & \; & \;\\ M_{i19}^{m} & M_{i20}^{m} & \cdots & M_{i18+n}^{m}\\ N_{i19}^{m} & N_{i20}^{m} & \cdots & N_{i18+n}^{m}\\ \; & \vdots & \; & \;\\ N_{i19}^{m} & N_{i20}^{m} & \cdots & N_{i18+n}^{m} \end{array}\right]A_{AL}^{ki}\left[\begin{array}{cccc} A_{i1}^{k1} & A_{i2}^{k1} & \cdots & A_{i20}^{k1}\\ \; & \vdots & \; & \;\\ A_{i1}^{km} & A_{i1}^{km} & \cdots & A_{i20}^{km} \end{array}\right]\\ \xi_{\text{EOP}}^{k}={\left[\begin{array}{llllll} dX_{C}^{k} & dY_{C}^{k} & dZ_{C}^{k} & d\omega^{k} & d\varphi^{k} & d\kappa^{k} \end{array}\right]}^{T}\\ \xi_{SP}^{i}={\left[\begin{array}{llllllllllll} da_{i0} & da_{i1} & da_{i2} & da_{i3} & db_{i0} & db_{i1} & db_{i2} & db_{i3} & dc_{i0} & dc_{i1} & dc_{i2} & dc_{i3} \end{array}\right]}^{T}\\ \xi_{t}^{i}={\left[\begin{array}{llll} dt_{i1} & dt_{i2} & \cdots & dt_{in} \end{array}\right]}^{T}\\ y^{ki}={\left[x_{p}^{ki}+f\frac{u^{0}}{w^{0}}y_{p}^{ki}+f\frac{u^{0}}{w^{0}}\text{Arc}{\left(t\right)}_{p}^{ki}-\text{Arc}{\left(t\right)}^{0}\right]}^{T}\\ \; \end{array}$$

with, $\text{Arc}{\left(t\right)}^{0}=\frac{t_{2}^{0}-t_{1}^{0}}{6}[f^{0}(t_{1}^{0})+4f^{0}\left(\frac{t_{2}^{0}+t_{1}^{0}}{2}\right)+f^{0}(t_{2}^{0})$, *m* as the number of images, *n* the number of points on a spline segment, *k* the *kth* image, and *i* the *ith* spline segment. Because the equation system of the integrated model has seven datum defects, the control information for the coordinate system is required to obtain seven transformation parameters. In a general photogrammetric network, the rank deficiency referred to as datum defects is seven. Estimates of the unknown parameters are obtained by the least squares solution, which minimizes the sum of squared deviations. A nonlinear least squares system is required in a conventional nonlinear photogrammetric solution to obtain orientation parameters. Many observations in photogrammetry are random variables that are considered as different values in the case of repeated observations such as the image coordinates of points. Each measured observation represents a random variable estimate. If image point coordinates are measured using a digital photogrammetric workstation, the values are measured slightly differently. The integrated and linearized Gauss–Markov model and the least squares estimated parameter vector with its dispersion matrix are:

(26) $$\begin{array}{l} y^{ki}=A_{IM}\xi_{IM}+e\\ A_{IM}=\left[\begin{array}{lll} A_{\text{EOP}}^{k} & A_{SP}^{i} & A_{t}^{i}\\ \; & A_{AL}^{ki} & \; \end{array}\right]\\ \xi_{IM}={\left[\begin{array}{ccc} \xi_{\text{EOP}}^{k} & \xi_{SP}^{i} & \xi_{t}^{i} \end{array}\right]}^{T}\\ {\hat{\xi }}_{IM}={\left(A_{IM}^{T}PA_{IM}\right)}^{-1}A_{IM}^{T}Py^{ki}\\ D\left({\hat{\xi }}_{IM}\right)=\sigma_{0}^{2}{\left(A_{IM}^{T}PA_{IM}\right)}^{-1} \end{array}$$

with $e∼N(0,\sigma_{0}^{2}P^{-1})$ being the error vector with zero mean and cofactor matrix *P*^−1^, a variance component $\sigma_{0}^{2}$, which can be known or not, *ξ̂_IM_* is the least squares estimated parameter vector, and *D*(*ξ̂_IM_*) is the dispersion matrix.

If one or more of the three estimated parameter sets $\xi_{\text{EOP}}^{k},\xi_{SP}^{i},\xi_{t}^{i}$ are considered as stochastic constraints, the reduction of the normal equation matrix can be applied. Control information is implemented as stochastic constraints in a bundle block adjustment. The distribution and quality of control features depend on the number and the density of control features, the number of tie features, and the degree of overlap of the tie features. If adding stochastic constraints removes the rank deficiency of the Gauss–Markov model, bundle adjustment can be implemented employing only the extended collinearity equations for the 3D natural cubic splines. Fixed exterior orientation parameters, control splines, or control spline location parameters can be stochastic constraints.

##### 3.5. Evaluation of Bundle Block Adjustment

Bundle block adjustment must be followed by an evaluation postadjustment analysis to check the suitability of project specifications and requirements. Iteratively reweighed least squares and least median of squares are the appropriate implementation of a statistical evaluation that removes poor observations. The important element affecting bundle block adjustment is the geometry of aerial images. Generally, the previous flight plan is adopted to obtain suitable results. A simulation bundle block adjustment is implemented before employing a flight plan within the new project design because such a simulation can reduce the effect of error measurements.

A qualitative evaluation that allows the operator to recognize the adjustment characteristics is often used after bundle block adjustment. The sizes of the residuals in images are drawn for the evaluation. The image residuals can be points or long lines and if all image residuals have the same orientation, then the image has a systematic error such as atmospheric refraction or an orientation parameter error. In addition, a lack of flatness in the focal plane may cause systematic errors in the image space, which affects the accuracy of a bundle block adjustment. Distortions are different from one location to another in the entire image space. The topographic measurement of the focal plane can correct the lack of focal plane flatness. Image coordinate errors are correlated in the case of systematic image errors. A poor measurement can result in an indicated opposite residual direction or an exaggerated residual.

The three main elements in the statistical evaluation of bundle block adjustments are precision, accuracy, and reliability. Precision is calculated employing parameter variances and covariances, because a small variance indicates that the estimated values have a small range and a large variance means that the estimated values are not calculated properly. The range of the parameter variance is from zero, in the case of error free parameters, to infinity, in the case of completely unknown parameters. A dispersion matrix may contain diagonal elements that are parameter variances. These and any off-diagonal elements are covariances between two parameters. Accuracy can be verified using check points that are not contained in bundle block adjustment like control points. Reliability can be confirmed from other redundant observations. The extended collinearity equations are a mathematical model for bundle block adjustment. The mathematical model consists of both functional and stochastic models. The functional one represents the geometrical properties and the stochastic one describes the statistical properties. Repeated measurements at the same location in the image space are represented with respect to the functional model and the redundant observations of image locations in the image space are expressed with respect to the stochastic model. While the Gauss–Markov model uses indirect observations, condition equations such as coordinate transformations and the coplanarity condition can be employed in the adjustment.

The Gauss–Markov model and the condition equation can be combined into the Gauss–Helmert model. In addition, functional constraints such as points having the same height or straight railroad segments can be added into the block adjustment.

The difference between condition and constraint equations is that condition equations consist of observations and parameters, and constraint equations consist of only parameters. With the advance of technology, the photogrammetrical input data has increased so adequate formulation of adjustment is required. All the variables are involved in the mathematical equations and the weight matrix of the variables changes from zero to infinity depending upon the variances. Variables with near to zero weight are considered as unknown parameters and variables with close to infinite weight are considered as constants. Most actual observations exist between the two boundary cases. Assessment by postadjustment analysis is important in photogrammetry to evaluate the results. One of the assessment methods is to compare the estimated variance with the two-tailed confidence interval based on the normal distribution. The two-tailed confidence interval is computed by a reference variance $\sigma_{0}^{2}$ with *χ*^2^ distribution as:

(27) $$\frac{r{\hat{\sigma }}_{0}^{2}}{\chi_{r,\alpha /2}^{2}}<\sigma_{0}^{2}<\frac{r{\hat{\sigma }}_{0}^{2}}{\chi_{r,1-\alpha /2}^{2}}$$

where *r* is degrees of freedom and *α* is a confidence coefficient (or a confidence level). If $\sigma_{0}^{2}$ has a value outside of the interval, we can assume that the mathematical model of adjustment is incorrect through the wrong formulation or linearization, blunders, or systematic errors.

##### 3.6. Pose Estimation with an ICP Algorithm

In the previous spline segment case, the correspondence between spline segments in the image and the object space was assumed. In the present consideration, it is not known which image points belong to which spline segment. The ICP algorithm can be utilized for the recovery of EOPs because the initial estimated parameters of the relative pose can be obtained from the orientation data for general photogrammetric tasks. The original ICP algorithm steps are as follows. The closest point operators search the associate point using the nearest neighboring algorithm and then the transformation parameters are estimated using a mean square cost function. The point is transformed by the estimated parameters and this step is iteratively established towards convergence into a local minimum of the mean square distance. The transformation, which includes translation and rotation between two clouds of points, is estimated iteratively towards convergence into a global minimum. In other words, the iterative calculation of the mean square errors is terminated when a local minimum falls below a predefined threshold. A small global minimum or a fluctuated curve requires more memory-intensive and time-consuming computation. In every iteration step, a local minimum is calculated with varying transformation parameters, but convergence into a global minimum with the correct transformation parameters is not always the result.

By the definition of a natural cubic spline, each parametric equation of a spline segment (*S_i_*(*t*)) can be expressed as:

(28) $$S_{i}(t)=\left[\begin{array}{c} X_{i}(t)\\ Y_{i}(t)\\ Z_{i}(t) \end{array}\right]=\left[\begin{array}{c} a_{i0}+a_{i1}t+a_{i2}t^{2}+a_{i3}t^{3}\\ b_{i0}+b_{i1}t+b_{i2}t^{2}+b_{i3}t^{3}\\ c_{i0}+c_{i1}t+c_{i2}t^{2}+c_{i3}t^{3} \end{array}\right],t\in [0,1]$$

with *X_i_*(*t*), *Y_i_*(*t*), *Z_i_*(*t*) as the object space coordinates and *a_i_, b_i_, c_i_* as the coefficients of the *ith* spline segment.

The ray from the perspective center (*X_C_,Y_C_,Z_C_*) to the image point (*x_p_,y_p_*,–*f)* is:

(29) $$\Xi (l)=\left[\begin{array}{c} X(l)\\ Y(l)\\ Z(l) \end{array}\right]=\left[\begin{array}{c} X_{C}^{k}\\ Y_{C}^{k}\\ Z_{C}^{k} \end{array}\right]+\left[\begin{array}{c} d_{1}\\ d_{2}\\ d_{3} \end{array}\right]l$$

where:

(30) $$\left[\begin{array}{c} d_{1}\\ d_{2}\\ d_{3} \end{array}\right]=R^{T}(\omega^{k},\varphi^{k},\kappa^{k})\left[\begin{array}{c} x_{p}\\ y_{p}\\ -f \end{array}\right]$$

with $X_{C}^{k},Y_{C}^{k},Z_{C}^{k},\omega^{k},\varphi^{k},\kappa^{k}$ EOPs at the *kth* iteration.

A point on the ray searches the closest to a natural cubic spline by minimizing the following target function for every spline segment. Transformation parameters related to an image point and its closest spline segment can be established using the least squares method:

(31) $$\Phi (l,t)\equiv {\left\|\Xi (l)-S_{i}(t)\right\|}^{2}=\text{stationary}\;l,t$$

The global minimum of Φ(*l, t*) can be calculated by ∇Φ(*l, t*) = 0 or ∂Φ/∂*l* = ∂Φ/∂*t* = 0. Substituting (28) and (29) into (31) and taking the derivatives with respect to *l* and *t* leads to:

(32) $$\begin{array}{l} \frac{1}{2}\frac{\partial \Phi }{\partial l}=\left(X_{C}+d_{1}l-a_{i0}-a_{i1}t-a_{i2}t^{2}-a_{i3}t^{3}\right)d_{1}+\left(Y_{C}+d_{2}l-b_{i0}-b_{i1}t-b_{i2}t^{2}-b_{i3}t^{3}\right)d_{2}+\left(Z_{C}+d_{3}l-c_{i0}-c_{i1}t-c_{i2}t^{2}-c_{i3}t^{3}\right)d_{3}=0\\ \frac{1}{2}\frac{\partial \Phi }{\partial l}=\left(X_{C}+d_{1}l-a_{i0}-a_{i1}t-a_{i2}t^{2}-a_{i3}t^{3}\right)\left(-a_{i1}-2a_{i2}t-3a_{i3}t^{2}\right)+\left(Y_{C}+d_{2}l-b_{i0}-b_{i1}t-b_{i2}t^{2}-b_{i3}t^{3}\right)\left(-b_{i1}-2b_{i2}t-3b_{i3}t^{2}\right)+\left(Z_{C}+d_{3}l-c_{i0}-c_{i1}t-c_{i2}t^{2}-c_{i3}t^{3}\right)\left(-c_{i1}-2c_{i2}t-3c_{i3}t^{2}\right)=0 \end{array}$$

Convergence into a global minimum does not exist because equation (32) is not a linear system in *l* and *t*. The relationship between an image space point and its corresponding spline segment cannot be established with the minimization method.

### 4. Experiments and Results

This section demonstrates the feasibility and the performance of the proposed model for the acquisition of spline parameters, spline location parameters, and image orientation parameters based on control and tie splines in the object space within the simulated and real data sets. In general photogrammetric tasks, the correspondence between image edge features must be established either automatically or manually, but in this study correspondence between image edge features is not required. In a series of six experiments with the synthetic data set, the first test recovers spline parameters and spline location parameters in an error free EOPs case. The second test recovers the partial spline parameters related to the spline shape. The third procedure estimates the spline location parameters with error free EOPs. The fourth step calculates EOPs and spline location parameters, followed by the fifth step that estimates EOPs with full controlled splines in which the parametric curves used as control features are assumed to be error free. In the last experiment, EOPs and tie spline parameters are obtained using the control spline.

**Figure 3.**
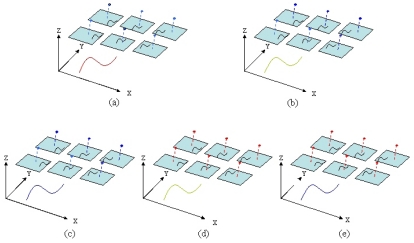
Different examples. (a) Known EOPs with tie splines, (b) Known EOPs with partial control splines, (c) Known EOPs with full control splines, (d) Unknown EOPs with partial control splines, and (e) Unknown EOPs with full control splines. (Red: Unknown parameters, Green: Partially fixed parameters, Blue: Fixed parameters).

Object space knowledge concerning splines, their relationships, and the orientation information of images can be considered as control information. Spline parameters in a partial control spline or orientation parameters can be considered as stochastic constraints in the integrated adjustment model. The starting point of a spline is considered to be a known parameter in the partial control spline in which *a*_0_,*b*_0_, and *c*_0_ of the *X, Y*, and *Z* coordinates of a spline are known. The number of unknowns is displayed in Table 1 and Figure 3, where *n* is the number of points in the object space, *t* shows the number of spline location parameters, and *m* represents the number of overlapped images in the target area.

Four points on a spline segment in one image are the only independent observations so additional points on the same segment do not provide nonredundant information to reduce the overall deficiency of the EOP and spline parameter recovery. To verify the information content of an image spline, we demonstrate that any five points on a spline segment generate a dependent set of extended collinearity equations. Any combination of four points yielding eight collinearity equations are independent observations, but five points bearing 10 collinearity equations produce a dependent set of observations related to the correspondence between a natural cubic spline in the image and the object space. More than four point observations on an image spline segment increase the redundancy related to the accuracy but do not decrease the overall rank deficiency of the proposed adjustment system. In the same fashion, the case using a polynomial of degree 2 can be implemented. Three points on a quadratic polynomial curve in one image are the only independent sets, so additional points on the same curve segment are a dependent observation. More than the independent point observations on a polynomial increase the redundancy related to the accuracy, but they do not provide nonredundant information.

The amount of information carried by a natural cubic spline can be calculated with the redundancy budget. Every spline segment has 12 parameters and every point measured on a spline segment contributes one additional parameter. Let *n* be the number of points measured on one spline segment in the image space and *m* be the number of images that contain a tie spline. 2*nm* collinearity equations and *m* (*n* − 1), the arc-length parameterizations, are equations and 12 (the number of one spline segment parameters) + *nm* (the number of spline location parameters) are unknowns. The redundancy is 2*nm* − *m* − 12 for one spline segment, so that if two images (*m* = 2) are used for bundle block adjustment, the redundancy is 4*n* − 14. Four points are required to determine spline and spline location parameters, in which case one spline segment and one degree of freedom to the overall redundancy budget is solved by each point measurement with the extended collinearity equation. Arc-length parameterization also contributes one degree of freedom to the overall redundancy budget. The fifth point does not provide additional information to reduce the overall deficiency but only strengthens the spline parameters. This means it increases the overall precision of the estimated parameters.

This fact shows the advantage of adopting splines in which the number of degrees of freedom is four because in straight tie lines only two points per line are independent. Independent information, the number of degrees of freedom of a straight line, is two from two points or a point with its tangent direction. A redundancy is *r* = 2*m* − 4 with a line expression of four parameters because there are 2 *nm* collinearity equations and the unknowns are 4 + *nm* [49]. Only two points (*n* = 2) are available to determine four line parameters with two images (*m* = 2) so at least three images must contain a tie line. The information content of *t* tie lines on m images is *t* (2*m* − 4). One straight line adds two degrees of freedom to the redundancy budget and at least three lines are required in the space resection. An additional point on a straight line does not provide additional information to reduce the rank deficiency of the recovery of EOPs but only contributes image line coefficients. If spline location parameters or spline parameters enter the integrated adjustment model through stochastic constraints, employing extended collinearity equations is enough to solve the system without the arc-length parameterization.

The redundancy budget of a tie point is *r* = 2*m* − 3 so tie points provide one more independent equation than the tie lines. However, using tie points requires a semiautomatic matching procedure to identify the tie points on all the images, and using linear features provides a more reliable and accurate basis for object recognition, pose determination, and other higher photogrammetric activities than using point features.

#### 4.1. Synthetic Data Description

To evaluate the new bundle block adjustment model using natural cubic splines, an analysis of the sensitivity and robustness of the model is required. The model suitability can be verified by using the estimated parameters with a dispersion matrix that includes standard deviations and correlations. The accuracy of bundle block adjustment is determined by the geometry of a complete block of images and the quality of the position and attitude information of a camera. A novel approach is a simulation of the bundle block adjustment. This is required prior to an actual experiment with real data in order to evaluate the performance of the proposed algorithms. Such a simulation can control the measurement errors to minimize random noise affecting the overall geometry of a block. Individual observations are generated based on the general situation of bundle block adjustment in order to estimate the properties of the proposed algorithms. A simulation allows adjustment for geometric problems or conditions with various experiments. A spline is derived via three ground control points (3232, 4261, 18), (3335, 4343, 52), and (3373, 4387, 34). Several factors that affect the estimates of exterior orientation parameters, spline parameters, and spline location parameters are discerned using the proposed bundle block adjustment model together with both the simulated image and the real image blocks.

#### 4.2. Experiments with Error Free EOPs

Spline parameters and spline location parameters are dependent upon various controls, and the unknowns can be obtained by a combined model of extended collinearity equations and the arc-length parameterization equations of splines. Splines in the object space are considered as tie lines in the same fashion as tie points in a conventional bundle block adjustment. Data on the exterior orientation parameters is considered as control information in this experiment. A well-known fact in employing the least squares system is that good initial estimates of true values make the system swiftly convergent towards the correct solution.

Normally distributed random noise is added to points in the image space coordinate system in all the experiments. This has a zero mean and *σ* = ±5*μm* standard deviation. Generally, the larger the noise level the more accurate are the approximations required to achieve the ultimate convergence of the results. A worst case scenario for estimation is that the large noise level causes the proposed model not to converge towards the specific estimates because the convergence radius is then proportional to the noise level. The parameter estimation is sensitive to the noise of the image measurement. Error propagation related to the noise in image space observation is one of the most important elements in the estimation theory. The proposed bundle block adjustment can be evaluated statistically using the variances and the covariances of parameters because a small variance indicates that the estimated values have a small range and a large variance means that the estimates are not properly calculated. The range of parameter variance is from zero in the case of error free parameters to infinity with completely unknown parameters. The result of one spline segment is expressed in Table 3 with *ξ*^0^ as the initial values and *ξ̂* as the estimates. The estimated spline and spline location parameters along with their standard deviations are established without the knowledge of the point-to-point correspondence.

If no random noise is added to image points, the estimates converge to the true values. The quality of initial estimates is important in the least squares system because it determines the iteration number of the system and the accuracy of the convergence. The assumption is that two points on one spline segment are measured in each image so the total number of equations is 2 × 6 (the number of images) × 2 (the number of points) + 6 (the number of the arc length), and the total number of unknowns is 12 (the number of spline parameters) + 12 (the number of spline location parameters). The redundancy (=the number of equations − the number of parameters), that is, the degrees of freedom, is six. While some of the geometric constraints such as slope and distance observations are dependent on the extended collinearity equations using splines, other constraints such as slope and arc length increase the nonredundant information in the adjustment to reduce the overall rank deficiency of the system.

The coplanarity approach is another mathematical model of the perspective relationship between the image and the object space features. The projection plane defined by the perspective center in the image space and the plane including the straight line in the object space are identical. Because the coplanarity condition is only for straight lines, the coplanarity approach cannot be extended to curves. Object space knowledge about the starting point of a spline can be employed in bundle block adjustment. Because the control information about a starting point is available for only three parameters of a total of 12 unknown parameters to a spline, a spline with control information about a starting point is called a partial control spline. Three spline parameters related to the starting point of a spline are set to stochastic constraints and the result is seen in Table 4. The total number of equations is 2 × 6 (the number of images) × 2 (the number of points) + 6 (the number of the arc length) = 30, and the total number of unknowns is 9 (the number of partial spline parameters) + 12 (the number of spline location parameters) = 21 so the redundancy is nine. A convergence of partial spline and spline location parameters has been archived with a partial control spline.

In the next experiment, spline location parameters are estimated with known EOPs and a full control spline. Because spline parameters and spline location parameters are dependent upon other parameters, the unknowns can be obtained from the model of an observation equation with stochastic constraints. In this experiment, spline parameters are set to stochastic constraints and the result is seen in Table 5.

The total number of equations is 2 × 6 (the number of images) × 3 (the number of points) = 36, and the total number of unknowns is 18 (the number of spline location parameters) so the redundancy is 18. Because spline location parameters are independent of each other, the arc-length parameterization is not required. The result indicates that a convergence of spline location parameters has been achieved with fixed spline parameters considered as stochastic constraints. The proposed model is robust with respect to the initial approximations of spline parameters. The uncertain information related to the representation of a natural cubic spline is described in the dispersion matrix.

#### 4.3. Recovery of EOPs and Spline Parameters

The object space knowledge of splines is available to recover the exterior orientation parameters in a bundle block adjustment. Control spline and partial control spline approaches are applied to verify the feasibility of using control information with splines. In both cases, equations of the arc-length parameterization are not necessary if we have enough equations to solve the system because spline parameters are independent of each other. In the experiment for a full control spline, the total number of equations is 2 × 6 (the number of images) × 4 (the number of points) + 3 (the number of arc lengths) × 6 (the number of images) = 66, and the total number of unknowns is 36 (the number of EOPs) + 24 (the number of spline location parameters) = 60. The redundancy is six. In the case of the partial control spline with one spline segment, the total number of equations is 2 × 6 (the number of images) × 4 (the number of points) + 3 (the number of arc lengths) × 6 (the number of images) = 66, and the total number of unknowns is 36 (the number of EOPs) + 9 (the number of partial spline parameters) + 24 (the number of spline location parameters) = 69. Thus, one more segment is required to solve the underdetermined system. The total number of equations using two spline segments is 2 × 6 (the number of images) × 4 (the number of points) × 2 (the number of spline segments) + 3 (the number of arc lengths) × 6 (the number of images) × 2 (the number of spline segments) = 132, and the total number of unknowns is 36 (the number of EOPs) + 9 (the number of partial spline parameters) × 2 (the number of spline segments) + 24 (the number of spline location parameters) × 2 (the number of spline segments) = 102. The redundancy is 30. A convergence of the EOPs of an image block and the spline parameters has been achieved in both experiments.

Table 6 expresses the convergence achievement of EOPs and spline location parameters. The correlation coefficient between parameter *X_C_* and *φ* is high (*ρ* ≈ 1) in the dispersion matrix, that is, two parameters are highly correlated among the EOPs. The correlation coefficient between parameters *Y_C_* and *ω* is approximately 0.85. In general, the correlation coefficient between parameters *X_C_* and *φ* is higher than between parameters *Y_C_* and *ω*.

Because a control spline provides the object space information about the coordinate system having datum defects of seven, tie spline parameters and EOPs can be recovered simultaneously. In the experiment of combined splines, the total number of equations is 2 × 6 (the number of images) × 3 (the number of points) × 2 (the number of splines) + 12 (the number of arc lengths) × 2 (the number of splines) = 96, and the total number of unknowns is 36 (the number of EOPs) + 12 (the number of tie spline parameters) + 18 (the number of tie spline location parameters) + 18 (the number of control spline location parameters) = 84.

Knowledge of object space information about a spline referred to as a full control spline is available prior to aerial triangulation. A control spline is considered to be a stochastic constraint in the proposed adjustment model and the representation of a control spline is the same as that of a tie spline. The result for combined splines that demonstrates the feasibility of using tie splines and control splines for bundle block adjustment is illustrated in Table 7.

Iteration with an incorrect spline segment in which a spline in the image space does not lie on the projection of a 3D spline in the object space results in a divergence of the system. A control spline is taken to be error free, but in reality this assumption is not correct. The accuracy of control splines is propagated into the proposed bundle block adjustment algorithm, but initial data such as a GIS database, maps, or orthophotos cannot be without error.

#### 4.4. Tests with Real Data

In this section, actual experiments with real data are undertaken to verify the feasibility of the proposed bundle block adjustment algorithm using splines for the recovery of EOPs and spline parameters. Medium scale aerial images covering the area of Jakobshavn Isbrae in West Greenland are employed for this study. The aerial photographs were obtained by Kort and Matrikelstyrelsen (KMS: Danish National Survey and Cadastre) in 1985. KMS established aerial triangulation using GPS ground control points with a ±1 pixel root mean square error under favorable circumstances and images were oriented to the WGS84 reference frame. Technical information on the aerial images is described in Table 8.

**Figure 6.**
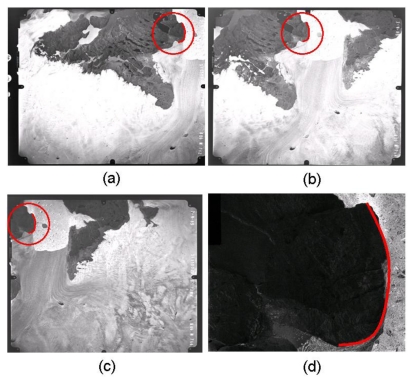
Test images. (a) Image 762, (b) Image 764, (c) Image 766, and (d) Target area.

The diapositive films were scanned with a RasterMaster photogrammetric precision scanner, which has a maximum image resolution of 12 μm and a scan dimension of 23 cm × 23 cm to obtain digital images for a softcopy workstation as seen in Figure 6.

The first experiment is the recovery of spline parameters with known EOPs obtained by manual operation using a softcopy workstation. A spline consists of four parts and the second segment parameters are recovered. The total number of equations is 2 × 3 (the number of images) × 3 (the number of points) + 2 (the number of arc lengths) × 3 (the number of images) = 24, and the total number of unknowns is 12 (the number of spline parameters) + 9 (the number of spline location parameters) = 21 so the redundancy is three. Table 9 shows the convergence achievement of spline and spline location parameters.

Estimation of spline parameters including their location parameters is established by the relationship between splines in the object space and their projection in the image space without the knowledge of the point-to-point correspondence. Because bundle block adjustment using splines does not require conjugate points generated by point-to-point correspondence knowledge, a more robust and flexible matching algorithm can be adopted. Table 10 shows the available object space information without knowledge of the point-to-point correspondence (the full control spline). All locations are assumed as lying on the second spline segment and the second spline segment as calculated from the softcopy workstation is used as control information.

The next experiment is the recovery of EOPs with a control spline. The spline control points are (534415.91, 767199305, −18.97), (535394.52, 7672045.02, 2.127), (536110.66, 7672024.29, −13.897), and (536654.04, 7671016.20, −2.51). Even though edge detectors are often used in digital photogrammetry and remote sensing software, the control points are extracted manually because edge detection is not our main goal. Among the three segments, the second spline segment is used for the EOP recovery. The information of the control spline is obtained by a manual operation using the softcopy workstation with an estimated accuracy of ±1 pixel. The convergence radius of the proposed iterative algorithm is proportional to the estimated accuracy level. The image coordinate system is converted into the photo coordinate system using the interior orientation parameters from KMS. The association between a point on a 3D spline segment and a point on a 2D image is not established in this study. Of course, 3D spline measurement in the stereo model using the softcopy workstation cannot be without error so the accuracy of the control spline is propagated into the recovery of EOPs. The result is illustrated in Table 11. The spline control information is utilized as stochastic constraints in the adjustment model. Because adding these constraints removes the rank deficiency of the Gauss–Markov model corresponding to spline parameters that are dependent upon spline location parameters, a bundle block adjustment can be made using only the extended collinearity equations for natural cubic splines.

## 5. Conclusions

In this paper, traditional least squares of a bundle block adjustment process have been augmented by support splines instead of conventional point features. Estimation of EOPs and spline parameters including location parameters is established by the relationship between splines in the object space and their projection into the image space without any knowledge of the point-to-point correspondence. Because bundle block adjustment using splines does not require conjugate points generated by the point-to-point correspondence knowledge, a more reliable and flexible matching algorithm can be adopted. Point-based aerial triangulation with experienced human operators is effective for traditional photogrammetric activities but is not appropriate within the autonomous environment of digital photogrammetry. Feature-based aerial triangulation is suitable for the development of reliable and accurate automation techniques. If linear features are employed as control features, they provide advantages over point features in aerial triangulation automation. Point-based aerial triangulation based on manual measurement and the identification of conjugate points is less reliable than feature-based aerial triangulation because it has the limitations of visibility (occlusion), ambiguity (repetitive patterns), and semantic information in the light of robust and appropriate automation. Automation of aerial triangulation and pose estimation is obstructed by the correspondence problem, but the employment of splines is one way to overcome occlusion and ambiguity issues. The manual identification of corresponding entities in two images is crucial in the automation of photogrammetric tasks. A further problem of point-based approaches is their weak geometric constraints as compared with feature-based methods, so accurate initial values for the unknown parameters are required. Feature-based aerial triangulation can be implemented without conjugate points because the measured points in each image are not the conjugate points in this proposed adjustment model. Thus, tie splines that do not appear in all the overlapped images together can be employed in feature-based aerial triangulation. Another advantage of employing splines is that the adoption of high level features increases the feasibility of geometric information and provides an appropriate analytical solution that emphasizes the redundancy of aerial triangulation.

3D linear features expressed by 3D natural cubic splines are employed as the mathematical model of linear features in the object space and its counterpart in the projected image space for bundle block adjustment. To solve overparameterization of 3D natural cubic splines, arc-length parameterization using Simpson's rule is developed, and in the case of straight lines and conic sections, spline tangents can be additional equations to the overparameterized system. Photogrammetric triangulation by the proposed model, including the extended collinearity and arc-length parameterization equations, is developed to show the feasibility of tie and control splines for the estimation of the exterior orientation of multiple images, splines, and spline location parameters. A useful stochastic constraint for a spline segment is examined for its utility to become a full or partial control spline such as known EOPs with a tie, partial control, and full control spline, and unknown EOPs with a partial and full control spline. In addition, the information content of an image spline is calculated and the feasibility of a tie spline and a control spline for a block adjustment is described. A simulation bundle block adjustment is implemented prior to the actual experiment with real data in order to evaluate the performance of the proposed algorithms. A simulation can control the measurement errors so that random noises minimally affect the overall geometry of a block. The individual observations are generated based on the general situation of bundle block adjustment to estimate the properties of the proposed algorithms. A simulation allows adjustment for geometric problems or varying conditions within individual experiments.

## Figures and Tables

**Figure 2.**
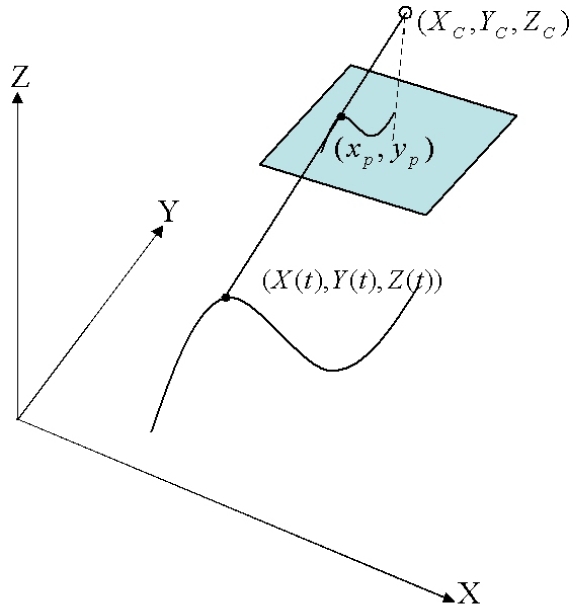
The projection of a point on a spline.

**Figure 4.**
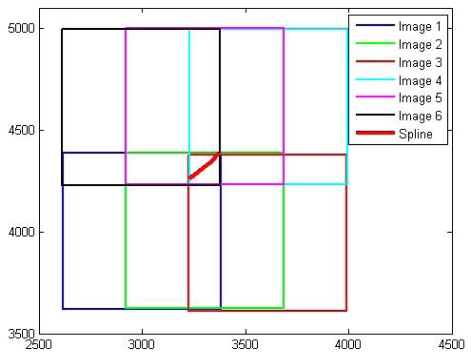
Six image block.

**Figure 5.**
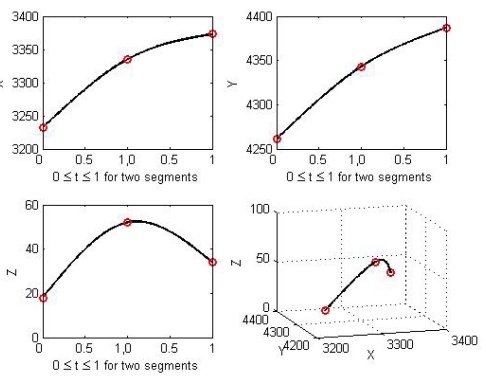
Natural cubic spline.

**Table 1.** Number of unknowns.

**EOP**	**Spline**	**Number of unknowns**
Known EOP	Tie spline	12(*n*–1) + *t*
	Partial control spline	9(*n*–1) + *t*
	Full control spline	*t*
Unknown EOP	Partial control spline	6*m* + 9(*n*–1) + *t*
	Full control spline	6*m* + *t*

**Table 2.** EOPs of six bundle block images for simulation.

**Parameter**	*X_C_*[*m*]	*Y_C_*[*m*]	*Z_C_*[*m*]	*ω* [deg]	*φ* [deg]	*κ* [deg]
Image 1	3000.00	4002.00	503.00	0.1146	0.0573	5.7296
Image 2	3305.00	4005.00	499.00	0.1432	0.0859	−5.7296
Image 3	3610.00	3995.00	505.00	0.1719	0.4584	2.8648
Image 4	3613.00	4613.00	507.00	0.2865	−0.0573	185.6383
Image 5	3303.00	4617.00	493.00	−0.1432	0.4011	173.0333
Image 6	2997.00	4610.00	509.00	−0.1833	−0.2865	181.6276

**Table 3.** Spline parameter and spline location parameter recovery.

Spline location parameters						
	Image 1		Image 2		Image 3	
	*t*_1_	*t*_7_	*t*_2_	*t*_8_	*t*_3_	*t*_9_
*ξ*^0^	0.02	0.33	0.09	0.41	0.16	0.47
*ξ̂*	0.0415±0.0046	0.3615±0.0016	0.0917±0.0017	0.4158±0.0032	0.1412±0.0043	0.4617±0.0135
	Image 4		Image 5		Image 6	
	*t*_4_	*t*_10_	*t*_5_	*t*_11_	*t*_6_	*t*_12_
*ξ*^0^	0.18	0.51	0.25	0.52	0.33	0.57
*ξ̂*	0.2174±0.0098	0.4974±0.0079	0.2647±0.0817	0.5472±0.0317	0.3133±0.0127	0.6157±0.1115
Spline parameters						
	*a*_10_	*a*_11_	*a*_12_	*a*_13_	*a*_10_	*a*_11_
*ξ*^0^	3322.17	72.16	−45.14	27.15	4377.33	69.91
*ξ̂*	3335.0080 ±0.0004	70.4660 ±0.0585	−48.8529 ±0.8310	16.5634 ±1.2083	4343.0712 ±0.0004	63.0211 ±0.0258
	*b*_12_	*b*_13_	*c*_10_	*c*_11_	*c*_12_	*c*_13_
*ξ*^0^	−17.49	13.68	48.82	10.15	–27.63	21.90
*ξ̂*	−28.7770 ±0.2193	9.8893 ±0.2067	51.9897 ±0.0006	8.1009 ±0.0589	−39.3702 ±0.7139	13.3904 ±1.0103

**Table 4.** Partial spline parameter and spline location parameter recovery.

Spline location parameters						
	Image 1		Image 2		Image 3	
	*t*_1_	*t*_7_	*t*_2_	*t*_8_	*t*_3_	*t*_9_
*ξ*^0^	0.04	0.36	0.09	0.40	0.14	0.45
*ξ̂*	0.0525±0.0067	0.3547±0.0020	0.1128±0.0047	0.4157±0.0091	0.1575±0.0028	0.4543±0.0083
	Image 4		Image 5		Image 6	
	*t*_4_	*t*_10_	*t*_5_	*t*_11_	*t*_6_	*t*_12_
*ξ*^0^	0.21	0.50	0.27	0.54	0.31	0.61
*ξ̂*	0.1916±0.0037	0.5128±0.0087	0.2563±0.0044	0.5319±0.0056	0.2961±0.0139	0.6239±0.1147
Spline parameters						
	*a*_11_	*a*_12_	*a*_13_	*b*_11_	*b*_12_	*b*_13_
*ξ*^0^	75.14	−52.87	30.71	70.05	−40.33	10.98
*ξ̂*	71.7099 ±0.0795	−47.2220 ±0.6872	−15.8814 ±2.6439	62.3703 ±0.0579	−28.7260 ±0.6473	7.1137 ±1.7699
	*c*_11_	*c*_12_	*c*_13_			
*ξ*^0^	0.82	−30.72	10.51			
*ξ̂*	7.1198 ±0.9483	−35.3841 ±1.3403	8.1557 ±3.5852			

**Table 5.** Spline location parameter recovery.

Spline location parameters						
	Image 1			Image 2		
	*t*_1_	*t*_7_	*t*_13_	*t*_2_	*t*_8_	*t*_14_
*ξ*^0^	0.01	0.37	0.63	0.09	0.44	0.71
*ξ̂*	0.0589±0.0015	0.3570±0.0076	0.6712±0.0197	0.1134±0.0072	0.4175±0.0054	0.7069±0.0080
	Image 3			Image 4		
	*t*_3_	*t*_9_	*t*_15_	*t*_4_	*t*_10_	*t*_16_
*ξ*^0^	0.17	0.46	0.74	0.21	0.49	0.81
*ξ̂*	0.1757±0.0031	0.4784±0.0071	0.7631±0.0095	0.2039±0.0102	0.4869±0.0030	0.8122±0.0044
	Image 5			Image 6		
	*t*_5_	*t*_11_	*t*_17_	*t*_6_	*t*_12_	*t*_18_
*ξ*^0^	0.26	0.53	0.84	0.29	0.61	0.89
*ξ̂*	0.2544 ±0.0050	0.5554 ±0.0069	0.8597 ±0.0089	0.3151 ±0.0095	0.6284 ±0.0052	0.9013 ±0.0086

**Table 6.** EOP and spline location parameter recovery.

EOPs								
Parameter		*X_C_*[*m*]	*Y_C_*[*m*]	*Z*_C_[*m*]	*ω* [deg]	*φ* [deg]	*κ* [deg]	
Image 1	*ξ*^0^	3007.84	4001.17	501.81	8.7090	−9.7976	−12.5845	
	*ξ̂*	3001.5852 ±0.0154	4001.2238 ±0.0215	503.2550 ±0.1386	–0.8908 ±0.3895	0.3252 ±0.1351	6.0148 ±0.8142	
Image 2	*ξ*^0^	3308.17	4001.17	497.52	10.23	8.3144	−5.5004	
	*ξ̂*	3305.1962 ±0.3804	4004.9827 ±0.1785	501.2641 ±0.2489	−0.1247 ±0.0308	−0.5497 ±0.0798	−5.2858 ±0.4690	
Image 3	*ξ*^0^	3612.68	3993.37	506.32	5.2731	7.2581	−10.135	
	*ξ̂*	3611.8996 ±0.1226	3995.7891 ±0.0695	505.1299 ±0.0337	0.1486 ±0.4467	0.1192 ±0.0168	2.3372 ±0.0794	
Image 4	*ξ*^0^	3619.75	4612.78	506.88	6.2571	−5.3482	183.66	
	*ξ̂*	3612.7128 ±0.0258	4613.0145 ±0.01895	507.0654 ±0.0251	−0.0921 ±0.7485	−0.152 ±0.4505	184.5016 ±0.2289	
Image 5	*ξ*^0^	3301.84	4618.63	497.61	−6.1731	7.5182	187.7145	
	*ξ̂*	3302.8942 ±0.0467	4617.0538 ±0.0249	492.9424 ±0.0704	−0.6347 ±0.1413	0.2662 ±0.8006	171.9808 ±0.6445	
Image 6	*ξ*^0^	2999.59	4615.74	508.49	−7.1651	−4.8427	185.1057	
	*ξ̂*	2997.9827 ±0.0513	4610.1432 ±0.0249	509.2952 ±0.0401	−0.1360 ±0.5659	−0.1279 ±0.6225	183.1789 ±0.2271	
Spline location parameters								
	Image 1				Image 2			
	*t*_1_	*t*_7_	*t*_13_	*t*_19_	*t*_2_	*t*_8_	*t*_14_	*t*_20_
*ξ*^0^	0.04	0.28	0.52	0.76	0.08	0.32	0.56	0.80
*ξ̂*	0.0432 ±0.0033	0.2980 ±0.0012	0.5176 ±0.0039	0.7705 ±0.0077	0.0813 ±0.0082	0.3338 ±0.0041	0.5715 ±0.0039	0.8136 ±0.0069
	Image 3				Image 4			
	*t*_3_	*t*_9_	*t*_15_	*t*_21_	*t*_4_	*t*_10_	*t*_16_	*t*_22_
*ξ*^0^	0.12	0.36	0.60	0.84	0.16	0.40	0.64	0.88
*ξ̂*	0.01294 ±0.0036	0.3578 ±0.0092	0.6024 ±0.0046	0.8437 ±0.0079	0.1594 ±0.0115	0.4112 ±0.0057	0.6418 ±0.0029	0.9783 ±0.0037
	Image 5				Image 6			
	*t*_5_	*t*_11_	*t*_117_	*t*_23_	*t*_6_	*t*_12_	*t*_18_	*t*_24_
*ξ*^0^	0.20	0.44	0.68	0.92	0.24	0.48	0.72	0.96
*ξ̂*	0.2039 ±0.0057	0.4461 ±0.0125	0.6713 ±0.0080	0.9264 ±0.0061	0.2483 ±0.0085	0.4860 ±0.0073	0.7181 ±0.0084	0.9613 ±0.0079

**Table 7.** EOP, control, and tie spline parameter recovery.

EOPs									
Parameter		*X*_C_[*m*]	*Y*_C_[*m*]	*Z*_C_[*m*]	*ω* [deg]	*φ* [deg]	*κ* [deg]		
Image 1	*ξ*^0^	3014.87	4007.18	500.79	0.9740	−8.6517	7.2155		
	*ξ̂*	3000.5917 ±0.0011	4001.8935 ±0.0059	503.2451 ±0.1572	−0.0974 ±0.1432	0.4297 ±0.0974	6.6005 ±0.2807		
Image 2	*ξ*^0^	3315.37	4008.57	503.31	−8.4225	−3.3232	7.2766		
	*ξ̂*	3305.1237 ±0.0057	4005.0571 ±0.0043	498.8916 ±0.0784	−0.5214 ±0.3610	−0.1948 ±0.1375	−6.1421 ±0.5558		
Image 3	*ξ*^0^	3613.85	3991.17	508.37	−1.3751	5.3783	4.3148		
	*ξ̂*	3609.5400 ±0.1576	3995.1419 ±0.0803	505.1791 ±0.0428	4.5378 ±5.4947	1.1746 ±0.3610	2.2288 ±0.4870		
Image 4	*ξ*^0^	3618.46	4617.61	503.18	8.5541	2.4287	182.7735		
	*ξ̂*	3613.1988 ±0.0599	4612.8281 ±0.0206	507.2056 ±0.0472	1.1803 ±0.2578	–0.4068 ±0.2979	185.7014 ±0.1089		
Image 5	*ξ*^0^	3305.71	4620.37	491.17	−8.7148	−5.1487	183.1114		
	*ξ̂*	3302.9716 ±0.0718	4617.0808 ±0.0592	492.9357 ±0.0660	−0.6990 ±0.1087	1.0485 ±0.1437	172.8671 ±0.2137		
Image 6	*ξ*^0^	3002.72	4613.63	491.22	8.5475	5.0124	178.2353		
	*ξ̂*	2996.9737 ±0.0315	4610.8773 ±0.0672	509.3724 ±0.0027	−3.2888 ±0.0688	0.5672 ±0.3837	182.2693 ±0.2478		
Control spline location parameters									
	Image 1			Image 2			Image 3		
	*t*_1_	*t*_7_	*t*_13_	*t*_2_	*t*_8_	*t*_14_	*t*_3_	*t*_9_	*t*_15_
*ξ*^0^	0.04	0.36	0.66	0.11	0.41	0.71	0.16	0.46	0.76
*ξ̂*	0.0597 ±0.0173	0.3495 ±0.0085	0.6518 ±0.0065	0.0982 ±0.0074	0.4085 ±0.0096	0.7087 ±0.0067	0.1494 ±0.0094	0.4499 ±0.0089	0.7564 ±0.0156
	Image 4			Image 5			Image 6		
	*t*_4_	*t*_10_	*t*_16_	*t*_5_	*t*_11_	*t*_17_	*t*_6_	*t*_12_	*t*_18_
*ξ*^0^	0.19	0.51	0.79	0.24	0.54	0.86	0.29	0.59	0.91
*ξ̂*	0.2018 ±0.0043	0.4984 ±0.0078	0.8065 ±0.0096	0.2573 ±0.0086	0.5586 ±0.0068	0.8553 ±0.0110	0.3172 ±0.0088	0.6137 ±0.0078	0.8958 ±0.0085
Tie spline location parameters									
	Image 1			Image 2			Image 3		
	*t*_1_	*t*_7_	*t*_13_	*t*_2_	*t*_8_	*t*_14_	*t*_3_	*t*_9_	*t*_15_
*ξ*^0^	0.03	0.34	0.67	0.09	0.39	0.71	0.14	0.47	0.73
*ξ̂*	0.0680 ±0.0073	0.3577 ±0.0067	0.6694 ±0.0033	0.1141 ±0.0116	0.4032 ±0.0073	0.6937 ±0.0054	0.1495 ±0.0124	0.4599 ±0.0075	0.7618 ±0.0054
	Image 4			Image 5			Image 6		
	*t*_4_	*t*_10_	*t*_16_	*t*_5_	*t*_11_	*t*_17_	*t*_6_	*t*_12_	*t*_18_
*ξ*^0^	0.21	0.49	0.81	0.26	0.56	0.83	0.31	0.58	0.92
*ξ̂*	0.1975 ±0.0026	0.5109 ±0.0019	0.8068 ±0.0216	0.2488 ±0.0773	0.5733 ±0.0027	0.8527 ±0.0138	0.3308 ±0.0034	0.6142 ±0.0115	0.9018 ±0.0317
Tie spline parameters									
	*a*_10_	*a*_11_	*a*_12_	*a*_13_	*b*_10_	*b*_11_			
*ξ*^0^	3341.44	73.13	−48.32	−20.72	4337.49	56.97			
*ξ̂*	3335.0147 ±0.0012	71.2914 ±0.0478	−47.5124 ±0.7959	−14.8527 ±1.8668	4342.0369 ±0.0009	62.4762 ±0.0804			
	*b*_12_	*b*_13_	*c*_10_	*c*_11_	*c*_12_	*c*_13_			
*ξ*^0^	−36.55	2.57	44.16	3.65	−28.22	7.99			
*ξ̂*	−28.0982 ±0.4851	6.8679 ±1.4219	51.5228 ±0.0008	6.8220 ±0.0421	−36.9681 ±1.9215	13.0338 ±2.0048			

**Table 8.** Information about aerial images used in this study.

Vertical aerial photograph	
Data	9 July 1985
Origin	KMS
Focal length	87.75 mm
Photo scale	1:150,000
Pixel size	12 μm
Scanning resolution	12 μm
Ground sampling distance	1.9 m

**Table 9.** Spline parameter and spline location parameter recovery.

Spline location parameters						
	Image 762			Image 764		
	*t*_1_	*t*_4_	*t*_7_	*t*_2_	*t*_5_	*t*_8_
*ξ*^0^	0.08	0.38	0.72	0.22	0.53	0.82
*ξ̂*	0.0844±0.0046	0.4258±0.0058	0.6934±0.0072	0.2224±0.0175	0.5170±0.0104	0.8272±0.0156
	Image 766					
	*t*_3_	*t*_6_	*t*_9_			
*ξ*^0^	0.32	0.59	0.88			
*ξ̂*	0.3075±0.0097	0.6176±0.0148	0.9158±0.0080			
Spline parameters						
	*a*_10_	*a*_11_	*a*_12_	*a*_13_		
*ξ*^0^	535000.00	830.00	−150.00	50.00		
*ξ̂*	535394.1732±0.1273	867.6307±0.7142	−173.1357±7.6540	24.3213±21.3379		
	*b*_10_	*b*_11_	*b*_12_	*b*_13_		
*ξ*^0^	7671000.00	150.00	140.00	−300.00		
*ξ̂*	7672048.3173±0.2237	143.1734±1.6149	130.8147±10.9058	−290.1270±26.7324		
	*c*_10_	*c*_11_	*c*_12_	*c*_13_		
*ξ*^0^	0.00	−10.00	−50.00	50.00		
*ξ̂*	2.1913±0.0547	−3.7669±0.1576	−39.8003±9.1572	27.7922±19.6787		

**Table 10.** Spline location parameter recovery.

Spline location parameters						
	Image 762		Image 764		Image 766	
	*t*_1_	*t*_4_	*t*_2_	*t*_5_	*t*_3_	*t*_6_
*ξ*^0^	0.15	0.60	0.30	0.75	0.45	0.90
*ξ̂*	0.1647±0.0048	0.6177±0.0091	0.2872±0.0034	0.7481±0.0093	0.4362±0.0155	0.9249±0.0087

**Table 11.** EOP and spline location parameter recovery.

EOPs							
Image		*X*_C_[*m*]	*Y*_C_[*m*]	*Z*_C_[*m*]	*ω* [deg]	*φ* [deg]	*κ* [deg]
762	*ξ*^0^	547000.00	7659000.00	14000.00	3.8472	2.1248	91.8101
	*ξ̂*	547465.37 ±15.0911	7658235.41 ±13.0278	13700.25 ±5.4714	0.3622 ±0.8148	0.5124 ±0.1784	91.5124 ±0.1717
764	*ξ*^0^	546500.00	7670000.00	13500.00	0.1125	0.6128	90.7015
	*ξ̂*	546963.22 ±12.5460	7672016.87 ±17.1472	13708.82 ±7.1872	−0.3258 ±0.6913	−0.5217 ±0.8632	91.1612 ±1.1004
766	*ξ*^0^	546000.00	768000.00	13700.00	1.4871	5.9052	92.0975
	*ξ̂*	546547.58 ±13.8104	7685836.75 ±12.1486	13712.20 ±8.4854	1.2785 ±1.4218	0.5468 ±1.1957	92.9796 ±0.6557

Spline location parameters						
	Image 762				Image 764	
	*t*_1_	*t*_4_	*t*_7_	*t*_10_	*t*_2_	*t*_5_
*ξ*^0^	0.08	0.32	0.56	0.80	0.16	0.40
*ξ̂*	0.0865±0.0097	0.3192±0.0159	0.5701±0.0072	0.8167±0.0088	0.1759±0.0067	0.4167±0.0085
	Image 764		Image 766			
	*t*_8_	*t*_11_	*t*_3_	*t*_6_	*t*_9_	*t*_12_
*ξ*^0^	0.64	0.88	0.24	0.48	0.72	0.96
*ξ̂*	0.6557±0.0131	0.8685±0.0092	0.2471±0.0086	0.4683±0.0069	0.7251±0.0141	0.9713±0.0089
